# SSX addiction in melanoma propagates tumor growth and metastasis

**DOI:** 10.3389/fonc.2022.998000

**Published:** 2022-10-07

**Authors:** Sofie Traynor, Malene Laage Ebstrup, Odd Lilleng Gammelgaard, Behzad Mansoori, Mikkel Green Terp, Cecilie Rose Hauge Rein, Sofie Rattenborg, Christina Bøg Pedersen, Henrik Jørn Ditzel, Morten Frier Gjerstorff

**Affiliations:** ^1^Department of Cancer and Inflammation Research, Institute of Molecular Medicine, University of Southern Denmark, Odense, Denmark; ^2^Department of Oncology, Odense University Hospital, Odense, Denmark; ^3^Academy of Geriatric Cancer Research (AgeCare), Odense University Hospital, Odense, Denmark

**Keywords:** melanoma, cancer/testis antigen (CTA), SSX, metastasis, cell cycle arrest

## Abstract

Cancer/testis antigens are receiving attention as targets for cancer therapy due to their germ- and cancer cell-restricted expression. However, many of these antigens are inconsistently expressed among cancer types and individual tumors. Here, we show that members of the SSX cancer/testis antigen family comprise attractive targets in the majority of melanoma patients, as SSX is expressed in more than 90% of primary melanomas and metastases and plays a critical role in metastatic progression. Accordingly, SSX silencing in melanoma mouse xenograft models reduced tumor growth and completely abolished the formation of metastatic lesions in lungs and livers. Mechanistically, we demonstrate that silencing SSX in melanoma cells induces cell cycle S-phase stalling, leading to proliferative arrest and enhanced apoptosis, which elucidates the inhibitory effect of SSX loss on tumor growth and colonization capacity. Silencing SSX further compromised the capacity of melanoma cells to migrate and invade, influencing these cells’ capability to spread and colonize. Taken together, these studies highlight SSX proteins as pivotal targets in melanoma with implications for blocking metastatic progression.

## Introduction

Cancer/testis antigens (CTAs) hold great potential as targets for cancer therapy due to their absence in healthy somatic tissues and frequent expression in many types of cancers (1). A major remaining challenge is to identify the best therapeutic targets among the hundreds of known CTAs. The Synovial Sarcoma, X-breakpoint (SSX) family of CTAs comprises six highly similar genes (i.e., SSX1, SSX2, SSX3, SSX4, SSX5 and SSX7) and several pseudogenes (2). Their expression correlates with advanced stages of disease and poor prognosis (1, 3–5), and multiple lines of evidence implicate SSX proteins in processes of tumor cell dissemination, such as epithelial-to-mesenchymal transition (EMT) and loss of focal adhesion (6–8).

Although the exact cellular role of SSX proteins has remained elusive, we and others have described SSX proteins as transcriptional repressors (9–12) and modulators of the activity of Polycomb repressive complexes (13, 14), which collectively suggest that SSX exert transcriptional control. Furthermore, the SSX-SYT fusion oncogene functionally rewires the SWI/SNF chromatin remodeling complex in synovial sarcoma (15), resulting in chromatin reorganization and oncogenic transformation (16). We have recently shown that SSX induces rearrangement and destabilization of heterochromatin in cancer cells, thereby promoting genomic instability (17). Together, these data implicate SSX proteins in deregulation of chromatin structure and induction of genomic instability, thereby supporting the development of cancer phenotypes.

Here, we demonstrate that SSX silencing in melanoma causes S-phase arrest and apoptosis, impeding the capacity of melanoma cells to form tumors, as well as inhibiting migration and invasion, compromising formation of distant metastases.

## Materials and methods

### Cell culture

FM6, FM79 and CBK14797 were kindly provided by Alexei Kirkin and Per Guldberg (The Danish Cancer Society). All cell lines were derived from metastatic tumors [info ESTDAB database or (18)]. cell lines were cultured in Roswell Park Memorial Institute (RPMI) 1640 medium (Sigma-Aldrich) supplemented with 10% fetal bovine serum (FBS, Sigma-Aldrich) and 1% penicillin/streptomycin. Cells were kept at 95% humidity and 5% CO_2_ as sub-confluent monolayers and were routinely tested for the presence of mycoplasma (Mycoalert, Lonza cat# LT070-118). Induction of SSX knockdown was induced by treatment with 250 ng/mL (FM79) or 500 ng/mL (FM6, CBK14797) doxycycline for 72 hours.

### Generation of melanoma cell lines with inducible knockdown of SSX

pZIP-TRE3G-ZsGreen plasmids with doxycycline induced expression of SSX target sequences: GCCTTCGATGATATTGCCAAAT and GCTGGTGATTTATGAAGAGATC in a miRNA scaffold was purchased from Transomic. The plasmids were prepared as lentiviral particles (17) and used for transduction of FM6, FM79 and CBK14797 cell lines. Stably transduced cells were selected using puromycin.

### TCGA data

Expression levels of the genes encoding members of the SSX family were examined in 103 primary melanoma and 368 metastatic melanoma samples from The Cancer Genome Atlas (TCGA) repository (https://www.cancer.gov/tcga, Project ID: TCGA-SKCM). RNA sequencing reads aligned to the Genome Reference Consortium Human Build 38 (GRCh38.p0) were downloaded and raw sequencing reads were normalized to library size as transcripts per million. Cluster analysis of SSX gene expression was performed using the Seurat V4 package in R (19).

Cluster analysis of SSX gene expression was performed using the Seurat V4 package in R. The 368 metastatic melanoma lesions were clustered into groups based on SSX1-5 expression levels using Uniform Manifold Approximation and Projection (UMAP) dimensional reduction.

### Western blotting

Cells were harvested in RIPA buffer and subjected to Western blotting using anti-beta-actin (Abcam Cat# ab6276, RRID : AB_2223210) and anti-SSX (OriGene Cat# TA502523, RRID : AB_11123843) primary antibodies. The secondary antibody used for enhanced chemiluminescent detection was horseradish peroxidase-conjugated rabbit anti-mouse IgG (Agilent Cat# P0260, RRID : AB_2636929).

### Crystal violet assay

Cells were seeded in 24-well plates and cultured with or without doxycycline for 72 hours. Cells were washed with PBS to discard dead cells and adherent cells were incubated with a crystal violet staining solution for 10 min at room temperature, followed by three washes in ddH_2_O and overnight drying. Cellular crystal violet was extracted by incubation with a citrate buffer for 30 min at room temperature on a shaker. Absorbance was measured at 570 nm using a Beckman Coulter PARADIGM Microplate Reader and SoftMax pro 7.0.2 software.

### Cell death detection assay

Apoptotic cell death was detected using the Cell Death Detection ELISA Kit (Roche, cat# 11774425001) according to manufacturer’s instructions. Briefly, cells were seeded at pre-determined cell densities in 24-well plates and cultivated with or without doxycycline for 72 hours. The media was discarded, and adherent cells resuspended in lysis buffer and incubated at room temperature for 30 min on a shaker. The plates were spun down at 200 x g for 10 min, and 20 µL of the supernatant was transferred to streptavidin-coated wells. An immunoreagent mix containing Biotin-labeled anti-histone antibodies, peroxidase-conjugated anti-DNA antibodies and incubation buffer was added to each sample. The plate was covered with adhesive foil and incubated on a shaker at room temperature for 2 hours. Thereafter, wells were washed three times with incubation buffer and ABTS solution added. Samples were incubated on a shaker at room temperature for 10-20 min until the color development was sufficient for photometric analysis, when ABTS stop solution was added. The absorbance was measured at 405 nm, with a reference wavelength of 490 nm, using a Beckman Coulter PARADIGM Microplate Reader and SoftMax pro 7.0.2 software.

### Propidium iodide (PI) staining

FM6, FM79, and CBK14797 cells were cultivated with or without doxycycline for 72 h. Following trypsinization, 1x10^6^ cells were transferred to a 15 mL tube, washed in ice-cold PBS and spun down at 300 x g for 5 min at 4°C. The supernatant was aspirated and 70% EtOH was added dropwise to the cells while vortexing, followed by overnight incubation at -20°C. Cells were spun down at 1000 rpm at 4°C for 5 min, the supernatant aspirated, and the cells washed in ice-cold PBS followed by centrifugation of 1000 rpm at 4°C for 5 min. The supernatant was removed, and the cell pellet resuspended in PBS containing DNase-free RNase A, followed by incubation at 37°C for 30 min. The cells were once again spun down at 1000 rpm at room temperature for 5 min, the supernatant was aspirated, and the cells resuspended in 0.3% Triton-X100/PBS with 20 µg/mL propidium iodide. The samples were incubated in the dark at room temperature for 20 min, then spun down at 1000 rpm at room temperature for 5 min and dissolved in PBS. Flow cytometric analysis was performed using a BD LSR II Flow Cytometer and BD FACSDiva Software. Cell Cycle analysis was performed using FlowJo 10.8.1.

### Wound healing assay

FM6-shScr, FM6-shSSX-A, FM6-shSSX-B, FM79-shScr, FM79-shSSX-A and FM79-shSSX-B cells were treated with vehicle or doxycycline for 48 hours to induce shRNA expression. The cells were seeded at pre-determined densities in 6-well tissue culture plates and incubated for 24 hours at 37°C and 5% CO_2_ to form confluent cell monolayers. Once reaching a 90-100% confluent monolayer, a linear wound was generated in the monolayers using a 200 μl pipette tip. Cellular debris was removed by washing with PBS, growth media was added, and initial images of the wound area were captured. Following incubation for 24 hours at 37°C with 5% CO_2,_ three representative images of the scratch area were captured. Results are presented as percentage of initial wound area width. The results were further normalized to cell proliferation using crystal violet staining of cells grown in parallel to the migration assay.

### Transmembrane migration assay

FM6-shScr, FM6-shSSX-A, FM6-shSSX-B, FM79-shScr, FM79-shSSX-A and FM79-shSSX-B cells were treated with vehicle or doxycycline for 48 hours to induce shRNA expression, followed by serum starvation for 24 hours. Cells (7.5x10^3^) in serum-free media were transferred to Matrigel-containing transwell inserts (Corning). Media containing serum was added to the bottom chamber and cells were allowed to migrate against the serum gradient for 24 hours at 37°C and 5% CO_2_. After 24 hours of incubation, non-migrated cells on the upper surface of the membrane were scraped off with a cotton applicator and the migrated cells on the bottom surface were fixed and stained with crystal violet for 10 min at room temperature. Excess dye was removed, and inserts were rinsed in H_2_O and air dried at room temperature before counting migrated cells. Cells in 5 random fields of view covering an area representing approximately 8-10% of the membrane surface were counted at 400x magnification. Cell counts were averaged and extrapolated for the entire area of the filter by multiplying by the ratio between the area of fields of view and the total area of the membrane. Percent migration was calculated by dividing migrated cells with the number of cells seeded. The results were further normalized to a concurrent proliferation assay determined by crystal violet staining.

### Animal studies

Mice were housed under pathogen-free conditions with *ad libitum* food and water. For tumor growth experiments, 1x10^6^ FM79-shScr (n = 16) or FM79-shSSX-A (n = 16) melanoma cells were injected subcutaneously into the right flank of female NOG CIEA mice (NOD.Cg-Prkdc^SCID^ ll2rg^tm1Sug^/JicTac, Tatonic). When tumors approached a size of 3-4 mm, the mice were randomized into two groups receiving 50 mg/ml doxycycline + 5% sucrose for induction of shRNA expression (n = 8) or 5% sucrose (control group, n = 8) in drinking water. When tumor size reached 12 mm, the mice were euthanized by cervical dislocation. Excised tumors were weighed and processed for immunohistochemical staining. For experimental metastasis experiments, FM79-shSSX-A cells were treated *in vitro* for three days with 250 ng/ml doxycycline or vehicle before one million cells were injected intravenously in the tail vein. Mice were continuously treated with 50 mg/ml doxycycline + 5% sucrose to maintain shRNA expression (n = 8) or 5% sucrose (control group, n = 8). After 5 weeks, mice were euthanized by cervical dislocation and lungs and livers were excised and processed for immunohistochemical staining. Mice experiments were approved by the Experimental Animal Committee of The Danish Ministry of Justice and were carried out in the core facility at the University of Southern Denmark.

### Immunohistochemical staining

Tissues were cut into 10 µM sections, which were deparaffinized and treated with 1.5% H_2_O_2_ in Tris-buffered saline (pH 7.5) for 10 min to block peroxidase activity. Sections were then washed in TNT buffer (0.1 M Tris, 0.15 M NaCl, 0.05% Tween-20, pH 7.5) and subjected to antigen retrieval using microwave boiling for 15 min in T-EG buffer (10 mM Tris, 0.5 mM EGTA, pH 9.0). Sections were then incubated with mouse anti-SSX (Sigma-Aldrich Cat# SAB1402364, RRID : AB_10640345) or mouse anti-Melan A (Ventana Medical Systems Cat# 790-2990, RRID : AB_2336015) diluted in antibody diluent (S2022, DAKO Cytomation, Glostrup, Denmark) for 1 hour at room temperature. Sections were then washed with TNT buffer and incubated with EnVision Flex/HRP+ for 30 min before washing and incubation with 3,3′-diaminobenzidine (DAB)+ substrate-chromogen for 10 min. Finally, samples were counterstained with Mayers hematoxylin before mounting in AquaTex (Merck Inc., Whitehouse Station, NJ, USA).

### Quantification of immunohistochemical staining

Immunohistochemical stainings of tumor, lung and liver sections were quantified using ImageJ. The relative levels of SSX and Melan-A positive cells were determined as the area of staining normalized to the total area of cells by adjusting the color threshold to mark all stained cells or all cells in the tissues.

### RNA extraction, cDNA synthesis, quantitative RT-PCR and RNA-seq

Cells were harvested in guanidium thiocyanate and RNA was purified according to the Chomczynski method (20). Purified RNA was analyzed by quantitative RT-qPCR or prepared for sequencing on the Illumina NovaSeq 6000 platform. For quantitative RT-PCR, 1 µg of RNA from three individual experiments was subjected to DNaseI treatment and cDNA was synthesized using random deoxynucleic acid hexameres and reverse transcriptase (Fermentas) according to manufacturer’s instructions. qRT-PCR was performed on the StepOne™ Real-Time PCR System (Applied Biosystems) using 2xSYBR mix (Applied Biosystems) according to the manufacturer’s instructions. All measurements were performed in triplicate. For determination of mRNA levels, exon-spanning primers were designed. The primer sequences are available upon request. For RNA sequencing, RNA from three independent experiments was prepared for sequencing on the Illumina NovaSeq 6000 platform using the NEBNext Poly(A) mRNA Magnetic Isolation Module (New England Biolabs, E7490L) and the NEBNext Ultra II DNA Library Prep Kit for Illumina (New England Biolabs, E7645L) with unique dual indexes according to manufacturer’s instructions.

### RNA-seq data analysis

The quality of raw sequencing reads was assessed using FASTQC (Babraham Bioinformatics) and adaptor sequences were removed using the FASTX toolkit. Trimmed Reads were aligned to the human genome (hg38) using the Spliced Transcripts Alignment to a Reference (STAR) software with default parameters (21). Tags in exons were counted using iRNA-seq (22) and differential expression (FDR-adjusted p-value ≤ 0.05) between three independent replicates of control and shSSX-B knockdown samples was determined using DESeq2 (23). Differentially expressed genes were defined as those having FDR ≤ 0.05 and a log2 fold change > 1.0 in either direction. Functional enrichment analysis to identify the biological processes and pathways altered by SSX knockdown was performed using HOMER (24).

### Statistical testing

Statistical analysis was carried out using two-tailed student’s *t*-tests or one-way ANOVA in the Prism 9.3.0 software. Statistical significance is indicated by asterisks: * = p < 0.05; ** = p < 0.01; *** < 0.001; **** < 0.0001.

## Results

### SSX genes are frequently expressed in melanoma primary tumors and metastases

**Figure 1:**
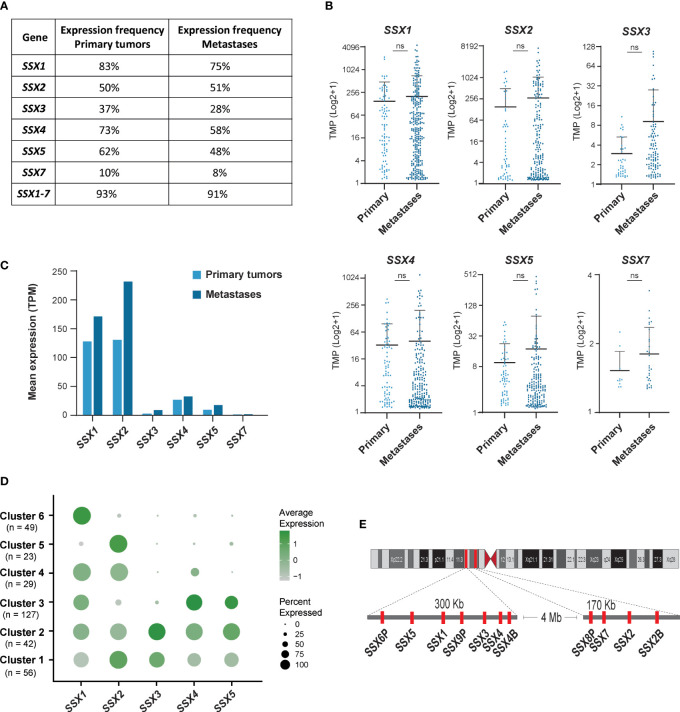
SSX genes are frequently expressed in primary melanomas and metastatic melanoma lesions. Large cohort analysis of SSX expression in primary melanomas (n = 103) and metastatic melanoma lesions (n = 368) using RNA-seq data from the TCGA repository. **(A)** Percentages of primary melanomas and metastatic melanoma lesions with SSX expression. SSX1-7 indicate the percentage of melanomas with detection of at least one SSX gene. **(B)** Expression levels of protein coding SSX genes in primary melanomas and metastatic melanoma lesions presented as transcripts per million (TPM). **(C)** Mean expression of SSX genes in melanoma primary tumors and metastatic melanomas. **(D)** Cluster analysis of SSX1-5 expression in 368 metastatic melanoma lesions demonstrating subsets of tumors with expression of different subsets of SSX genes. **(E)** Schematic representation of the two SSX gene clusters on chromosome X. ns, non-significant.

Earlier studies with small cohorts of patients suggested that SSX members are expressed in about 40% of melanoma tumors (2, 4, 25), but not in melanocytes and other normal tissues and only rarely in melanocytic nevi (8, 26) (data from The Human Protein Atlas; https://www.proteinatlas.org). We used RNA-sequencing data available from the TCGA repository to investigate the expression of individual SSX family members in a larger number of primary (n = 103) and metastatic (n = 368) melanomas. All six protein-coding members of the SSX gene family were detected in primary tumors and metastases, although SSX7 was expressed at very low levels (**Figures 1A–C**). At least one SSX gene was detectable in 93% of primary tumors and 91% of metastases, showing that SSX genes are more commonly expressed in melanoma than previously anticipated (**Figures 1A–C**). In accordance with previous small cohort studies, the highest occurrence and expression levels were observed for *SSX1* and *SSX2*, with 89% of primary melanomas and 88% of metastases having expression of either *SSX1*, *SSX2* or both (**Figure 1B**). Clustering analysis of the expression of *SSX1-5* in melanoma tumors (**Figure 1D**) showed that the majority (80%) of melanomas expressed more than one SSX gene, and that some SSX genes correlated in their expression despite being in different genomic loci (**Figure 1E**). Most notably, SSX2 was frequently coexpressed with SSX3 and SSX4 were often coexpressed with SSX5 (**Figure S1**). There was no association between melanoma stage and SSX expression, although there was a tendency towards higher expression with more advanced disease (**Figure S2**). Altogether, these results show that SSX members are frequently expressed in melanoma tumors with potential implications for melanoma therapy. Furthermore, our data reveal important expression patterns of individual SSX genes in melanoma, which should be taken into consideration when designing SSX-directed therapy.

### SSX silencing impedes melanoma tumor growth and metastasis

**Figure 2:**
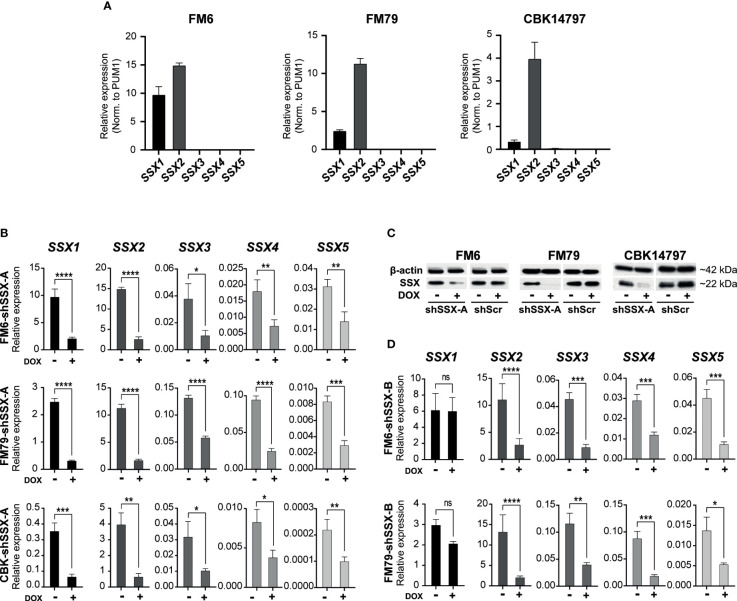
Melanoma cell lines resemble clinical melanoma tumors with respect to SSX expression. **(A)** Expression levels of SSX1-5 in FM6, FM79 and CBK14797 melanoma cell lines. Expression was determined using RT-qPCR with SSX isoform-specific primers and normalized to expression of PUM1 **(B)** Doxycycline induced lentiviral-mediated shRNA knock-down of SSX1-5 (shSSX-B). **(C)** Western blot analysis was used to assess protein levels of SSX in FM6, FM79 and CBK14797 cells with and without doxycycline treatment. A scrambled shRNA control was used as a control. βeta-actin was used as loading control. **(D)** Doxycycline induced lentiviral-mediated shRNA knockdown of SSX2-5 (shSSX-A). Error bars represent SD (*n* = 3). Statistical differences between samples were analyzed by t-test. (****) *P* < 0.0001; (***) *P* < 0.001; (**) *P* < 0.01; (*) *P* < 0.05; (ns) not significant (*P* > 0.05).

We investigated the role of SSX members in melanoma tumor growth and metastasis. To this end, we established three melanoma cell lines (FM6, FM79 and CBK14797) with doxycycline-induced knockdown of SSX expression (**Figure 2**). Resembling the clinical melanoma samples, all SSX genes could be detected in FM6, FM79 and CBK14797 before knockdown, but the expression was dominated by *SSX1* and *SSX2* (**Figure 2A**). Due to the expected functional redundancy among SSX members, the expression of all SSX genes was silenced using a short hairpin RNA (shSSX-A) targeting a conserved mRNA sequence (**Figures 2B, C**). A second shRNA (shSSX-B) targeting the most highly expressed SSX gene in our models, *SSX2*, as well as the structurally similar genes (i.e. *SSX3-5*) was also included in our analysis (**Figure 2D**).

**Figure 3:**
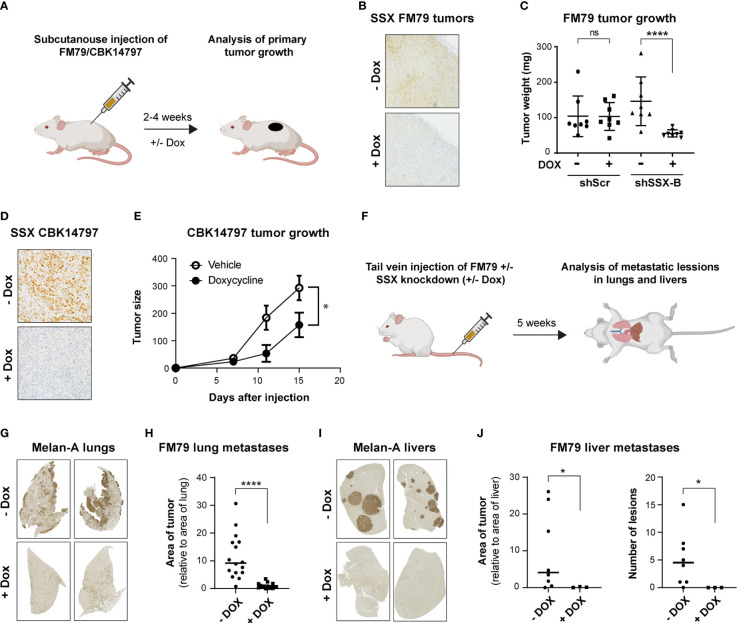
SSX silencing in melanoma cells inhibits tumor growth and metastasis. **(A)** Schematic representation of mouse models for evaluation of the role of SSX in tumor formation. CIEA NOG mice with established FM79 or CBK14797 tumors were treated with doxycycline (+Dox) in drinking water to induce intra-tumoral SSX knockdown and were compared to a control treatment group (-Dox). After 2-4 weeks, mice were terminated, and tumors were resected. n=8 per treatment group. Illustration was generated using Biorender. **(B, C)** FM79 tumor sections were stained for SSX to validate knockdown in doxycycline-treated tumors. Representative stainings are shown **(B)**. Tumor weight following resection of tumors from mice challenged with either FM79-shScr or FM79-shSSX-B cells and treated with doxycycline or vehicle **(C)**. **(D, E)** Mice challenged with CBK14797 tumors were treated with doxycycline to induce intra-tumoral SSX knockdown or vehicle. Tumor sections were stained for SSX to validate knockdown in doxycycline-treated tumors. Representative stainings are shown **(D)**. Tumor development in groups with and without doxycycline treatment **(E)**. **(F)** Schematic representation of mouse model for evaluation of the role of SSX in formation of experimental metastases. FM79-shSSX-B cells were treated *in vitro* with doxycycline or vehicle and subsequently injected in the tail vein of CIEA NOG mice. Mice were treated with vehicle or doxycycline in drinking water to maintain SSX expression or knockdown, respectively. After five weeks, mice were euthanized and lungs and livers were excised and processed for immunohistochemical staining of the melanocytic lineage marker Melan A to identify metastatic melanoma cells. Illustration was generated using Biorender. **(G-J)** Quantification of metastatic burden in the FM79 experimental metastases model. Representative Melan-A stainings of resected lungs **(G)**. Quantification of metastatic lesions in resected lungs. The area of Melan-A staining is normalized to the total area of the lungs **(H)**. Representative Melan-A stainings of resected livers **(I)**. Quantification of metastatic lesions in resected livers. The area of staining is normalized to the total area of the livers (J, left). Number of metastatic lesions in livers (J, right). Statistical differences between samples were analyzed by t-test. (****) *P* < 0.0001; (*) *P* < 0.05; (ns) not significant (*P* > 0.05).

The role of SSX genes in melanoma tumor growth *in vivo* was investigated using FM79 and CBK14797 melanoma cells (**Figure 3A**), which were able to form tumors in NOG mice. Cells stably transduced with the shRNA construct targeting all SSX genes (shSSX-A) or with a scrambled shRNA construct were implanted subcutaneously into the flank of mice. When the tumor diameter reached a size of 2-3 mm, mice were treated orally with doxycycline to induce shRNA expression or with vehicle as control. For both cell lines, knockdown of SSX expression significantly reduced tumor growth (**Figures 3B–E**).

Given the frequent expression of SSX genes in melanoma metastases and the association between SSX members and processes of tumor cell dissemination, such as epithelial-to-mesenchymal transition (EMT) and loss of focal adhesion (6–8), we also investigated the effect of SSX members on the ability of melanoma cells to form metastatic lesions in mice. None of the used melanoma cell lines were able to form spontaneous metastases (data not shown). To generate experimental metastasis, we injected FM79 cells with or without SSX knockdown in the tail vein of NOG mice (**Figure 3F**). Immunohistochemical analysis of lung and liver upon termination of the mice demonstrated a major reduction in tumor burden upon SSX knockdown (**Figures 3G–J**). Specifically, while all mice injected with SSX-expressing FM79 cells displayed extensive lung colonization, there were no metastatic nodules or single tumor cells in the lungs of mice that were injected with FM79 cells with SSX knockdown (**Figures 3G, H**). A similar pattern was seen in the livers of mice. While the livers of mice injected with SSX-expressing FM79 exhibited large metastatic nodules, neither metastatic nodules nor single tumor cells were observed in livers of mice with SSX knockdown cells (**Figures 3I, J**).

These data show that silencing SSX expression significantly reduced tumor growth of melanoma cells and completely compromised their ability to form experimental metastases.

### SSX silencing induces cell proliferation arrest and apoptosis

**Figure 4:**
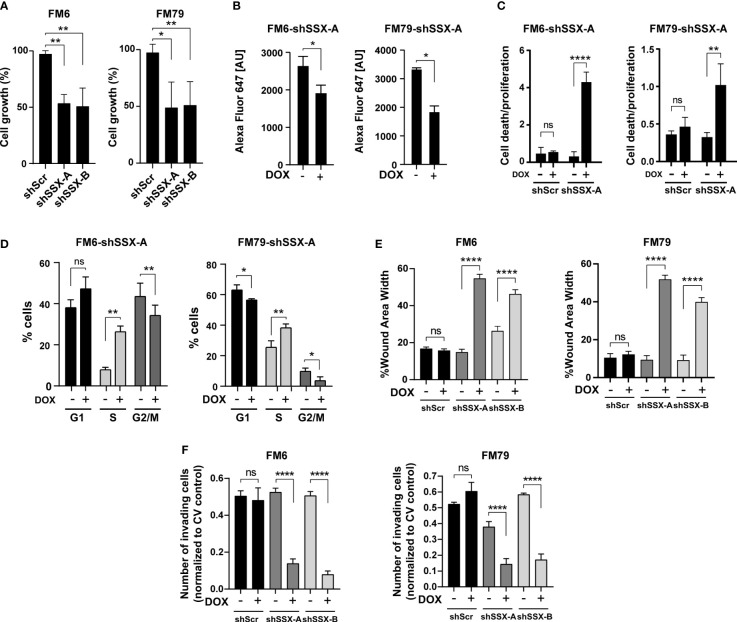
SSX silencing induces S-phase arrest and diminishes the invasive potential of melanoma cells. *In vitro* assays performed with FM6 and FM79 melanoma cells transduced with SSX-targeting shRNAs (shSSX-A and -B) or scrambled shRNA (shScr) **(A)** Cellular growth based on the relative number of cells quantified by crystal violet staining. **(B)** Relative quantification of replication using EdU labelling of newly synthesized DNA. The amount of incorporated EdU is shown as the geometric mean fluorescence intensity of Alexa-Fluor-647 [AU]. **(C)** Apoptotic cell death evaluated using a Cell Death Detection Elisa assay normalized to the relative amounts of cells determined by crystal violet staining. **(D)** Percentage-wise grouping of G0/G1-, S- and G2/M-phase cells based on flow cytometric quantification of propidium iodide-stained DNA. Cell cycle analysis was performed using the Dean-Jett-Fox (DJF) algorithm to model the cell cycle data. In **A–D**, cells were cultivated with or without doxycycline for 72 hours prior to experiments. **(E)** Quantification of wound area width 24 hours after wound generation for confluent cells. The results are normalized to proliferative capacity as determined by a concurrently performed proliferation assay. **(F)** Number of invading (Transwell) cells normalized to proliferative capacity determined by a concurrent proliferation assay. Error bars represent SD (*n* = 3). Statistical differences between samples were analyzed by t-test or one-way ANOVA followed by Tukey’s multiple comparison test. (****) *P* < 0.0001; (**) *P* < 0.01; (*) *P* < 0.05; (ns) not significant (*P* > 0.05).

To mechanistically understand how SSX silencing reduces melanoma growth and abolishes formation of experimental metastases, we studied the effect of SSX knockdown on cell growth and survival. SSX knockdown significantly reduced the growth (**Figure 4A**), proliferation as determined by DNA incorporation of EdU (**Figures 4B**, **S3**), and induced apoptosis (**Figure 4C**) of both FM6 and FM79 cells. Furthermore, cell cycle analysis demonstrated that cells arrested in S-phase in response to SSX knockdown (**Figures 4D**, **S3 **and **S4**). Taken together, these data strongly suggest that melanoma cells become addicted to SSX expression and that compromised cell cycle progression and increased apoptosis of melanoma cells with SSX knockdown account for the diminished growth of melanomas with reduced SSX levels (**Figures 3A–E**). They further indicate that SSX proteins are important for S-phase progression in melanoma cells.

### SSX silencing significantly impairs cell migration and invasion of melanoma cells

The frequent expression of SSX family members in melanoma metastases and complete blockade of metastases formation of FM79 cells upon SSX knockdown suggested a specific role for SSX in supporting processes of metastatic progression. To further investigate this, we measured the effect of SSX knockdown on migration and invasion of melanoma cells using wound healing and transmembrane migration assays. To exclude potential effects of SSX knockdown-mediated cell growth arrest and apoptosis, we only used serum-starved live cells for these experiments and adjusted results to changes in cell numbers during the experiments. Importantly, both the wound healing and transmembrane migration assays clearly demonstrated that FM6 and FM79 cells displayed reduced capability to migrate and invade following SSX knockdown (**Figures 4E, F** and **S4**). This was supported by RNA sequencing analysis showing that SSX knockdown reduces the expression of genes involved in focal adhesion, integrin-mediated cell adhesion and ECM-receptor interaction (**Figure S5**).

These data suggest that SSX supports the migration and invasion potential of melanoma cells. Together with the observed reduction in cell proliferation and increased rates of apoptosis upon SSX knockdown, this accounts for the reduced capacity of SSX-silenced FM79 cells to form metastatic lesions *in vivo* and supports a role for SSX family members in melanoma progression.

## Discussion

In this study, we investigated the therapeutic potential of targeting SSX expression in melanoma tumors. More than 90% of primary and metastatic melanomas expressed one or more SSX genes, highlighting their potential as targets. Despite the high structural similarity of SSX genes, our analysis revealed a high level of heterogeneity in the expression of SSX genes among melanomas. For instance, many tumors expressed only *SSX1* or *SSX2*, while others expressed different combinations of two or more SSX genes. Furthermore, *SSX1* and *SSX2* were generally expressed at higher levels than other SSX genes. These data suggest that therapeutic strategies targeting several SSX molecules, including *SSX1* and *SSX2*, may be effective for a broad segment of melanoma patients.

Interestingly, SSX silencing in melanoma cells resulted in potent reduction of tumor growth, supporting a rationale for targeting SSX in melanoma. *In vitro* studies further demonstrated that SSX silencing induced crisis in melanoma cell cultures leading to S-phase arrest and apoptosis. This is in line with previous studies from our group showing that changes in SSX expression perturbs cell cycle progression in S-phase (17, 27). In melanoma cells, overexpression of SSX2 was shown to induce instability of pericentromeric heterochromatin in S-phase, and in breast cancer cells, SSX2 overexpression induced a senescence response in a S-phase dependent manner (17, 27). Thus, it seems that SSX proteins play important roles in S-phase progression in cancer cells and, once SSX genes are activated, the cells become dependent on SSX to retain genome integrity and cell cycle progression. Although the precise mechanism of S-phase arrest in response to changes in SSX levels remains elusive, the documented role for SSX proteins in modifying chromatin structure suggest that significant changes in SSX protein levels may impede re-establishment of chromatin structure following replication and trigger S-phase checkpoints.

Our data also reveal that SSX loss significantly impairs the capacity of melanoma cells to form metastases in lungs and livers. The use of an experimental metastasis setup (i.e. intravenous injection of tumor cells), represents the last part of the metastatic process wherein cells extravasate into tissues and proliferate to form metastatic nodules. These processes may largely be affected by the documented effects on cell cycle progression and apoptosis by SSX knockdown, which may significantly reduce the capacity of cells to colonize tissues and form metastases. However, *in vitro* experiments also showed that SSX loss significantly impairs the capacity of melanoma cells to migrate, which is highly important for the invasive potential of cancer cells. This aligns with a previous study implicating SSX in beta-catenin signaling and epithelial-to-mesenchymal transition (6). Thus, SSX silencing may impair several aspects of the metastatic process.

In conclusion, we have demonstrated that targeting of SSX in melanoma cells severely compromises their growth and metastatic capacity, highlighting SSX proteins as promising therapeutic targets. This may lay the foundation for developing novel shRNA- or CRIPSR-based therapies targeting the expression of SSX or molecules functionally related to SSX to prevent metastatic spread of melanoma. The intratumoral heterogeneity in SSX expression (8) represents a potential challenge that needs to be addressed before the full therapeutic potential of targeting SSX molecules can be realized.

## Data availability statement

The original contributions presented in the study are included in the article/**Supplementary Material**. Further inquiries can be directed to the corresponding author.

## Ethics statement

The animal study was reviewed and approved by Experimental Animal Committee of The Danish Ministry of Justice.

## Author contributions

Conceptualization (ST and MG), experimental work (ST, ME, CR, SR, CP, BM, and OG), data analysis and visualization (ST, ME, and BM), writing - original draft (ST and MG), writing - review & editing (ST, MG, HD, OG, and MT), project administration (ST and MG), funding acquisition (MG, ST, and HD). All authors contributed to the article and approved the submitted version.

## Funding

This work was supported by Pink Tribute, Laege Sofus Carl Emil Friis og Hustru Olga Doris Friis Legat, Dagmar Marshalls Fond, the Danish Cancer Society, the Academy of Geriatric Cancer Research (AgeCare), the Novo Nordisk Foundation and the Danish Research Council for Independent Research.

## Acknowledgments

We thank the Animal Core Facility at University of Southern Denmark for animal care, Department of Pathology for technical assistance with immunohistochemistry and M. Kat Occhipinti for editorial assistance.

## Conflict of interest

The authors declare that the research was conducted in the absence of any commercial or financial relationships that could be construed as a potential conflict of interest.

## Publisher’s note

All claims expressed in this article are solely those of the authors and do not necessarily represent those of their affiliated organizations, or those of the publisher, the editors and the reviewers. Any product that may be evaluated in this article, or claim that may be made by its manufacturer, is not guaranteed or endorsed by the publisher.
