# Supplementary material for: SSX addiction in melanoma propagates tumor growth and metastasis
Source: Front Oncol. 2022 Oct 7;12:998000. doi: 10.3389/fonc.2022.998000 (PMC9585237; doi:10.3389/fonc.2022.998000)

**Figure S1. Coexpression of SSX genes in melanomas.** Pairwise correlation analysis of SSX expression. Correlation analysis was performed with TCGA melanoma gene expression data using Graphpad Prism.


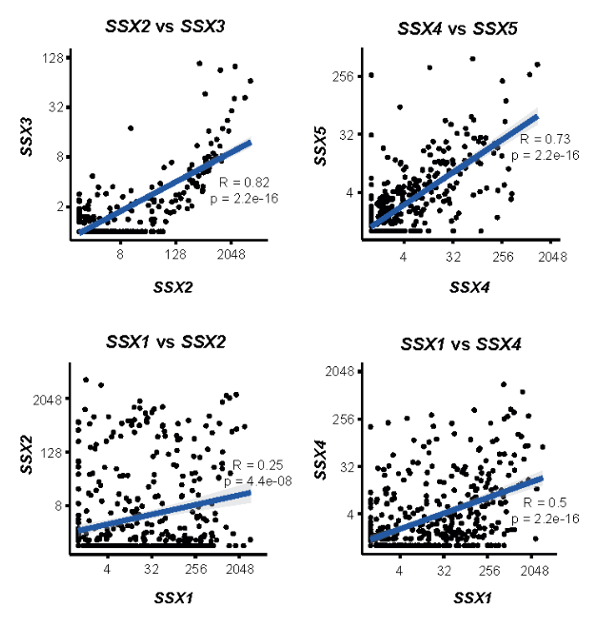


**Figure S2. Association between SSX expression and melanoma stage.** Data from a cohort of patients with melanoma (n = 144) [1] was analyzed and extracted using <http://www.cbioportal.org/>. Representation and statistical testing of data was done in GraphPad Prism 9.

1 Liu D, Schilling B, Liu D, Sucker A, Livingstone E, Jerby-Arnon L *et al*. Integrative molecular and clinical modeling of clinical outcomes to PD1 blockade in patients with metastatic melanoma. *Nat Med* 2019; 25: 1916-1927.

**
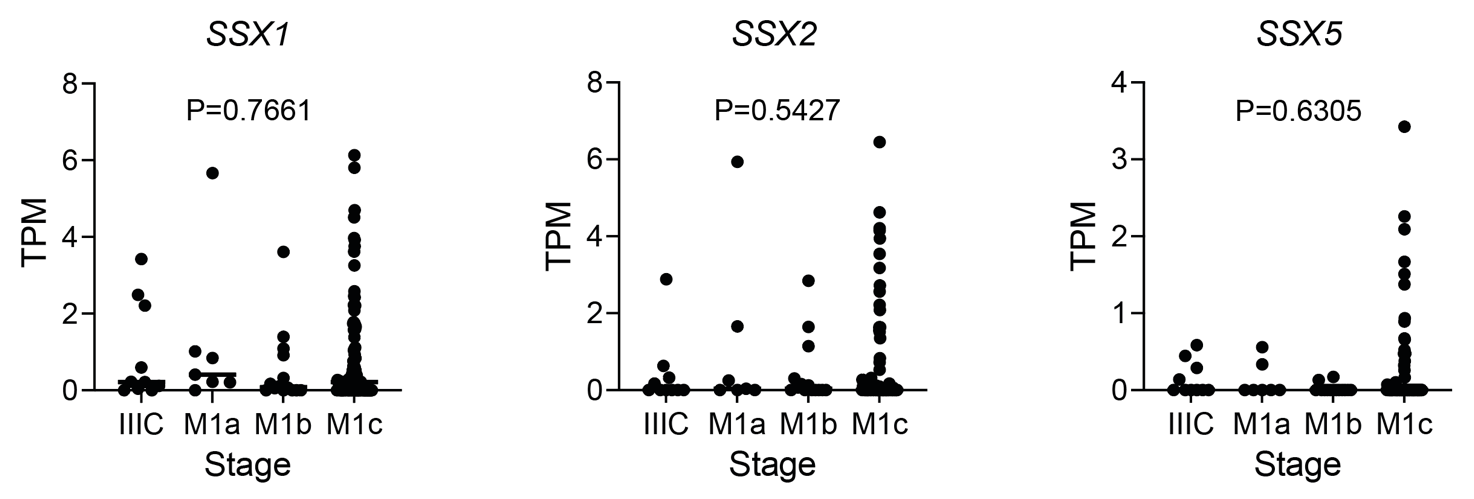
**

**Figure S3. Cell cycle and EdU analysis of FM79 cells transduced with scrambled shRNA** (**A**) Percentage-wise grouping of G0/G1-, S- and G2/M-phase cells based on flow cytometric quantification of propidium iodide-stained DNA in FM6-shScr and FM79-shScr cells with or without doxycycline treatment. Cell cycle analysis was performed using the Dean-Jett-Fox (DJF) algorithm to model the cell cycle data. (**B**) Relative quantification of replication in FM6-shScr and FM79shScr cells using EdU labelling of newly synthesized DNA. The amount of incorporated EdU is shown as Geometric mean fluorescence intensity of Alexa-Fluor-647 [AU]. Error bars represent SD (*n* = 3). Statistical differences between samples were analyzed by t-test. (ns) not significant (*P* > 0.05).


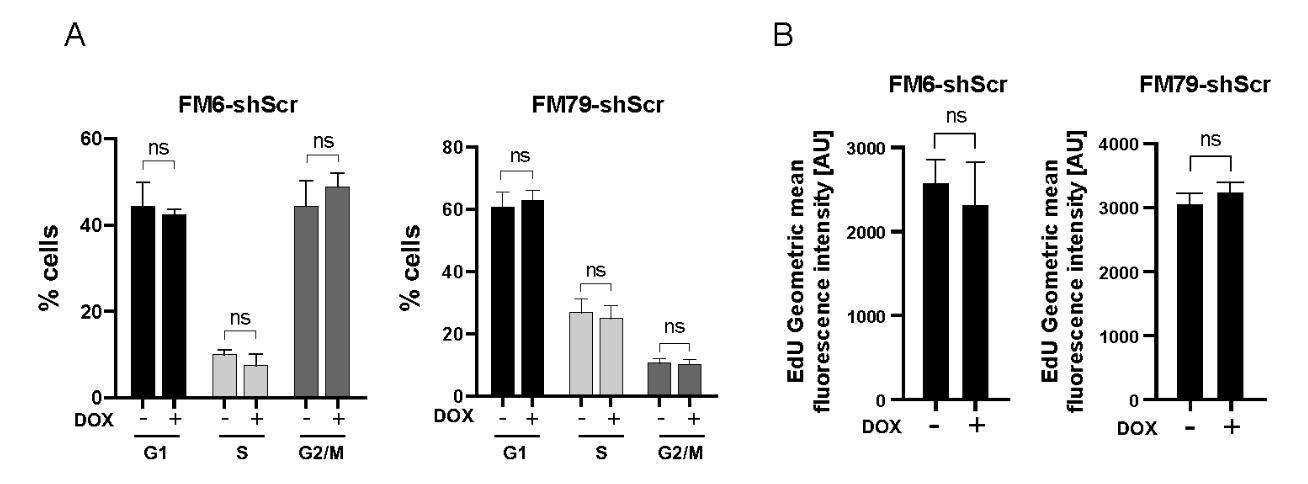


**Figure S4. Repressentative images of wound healing (A), transwell assays (B) and FACS analysis (C) conducted on shSSX and shSrc cells.**


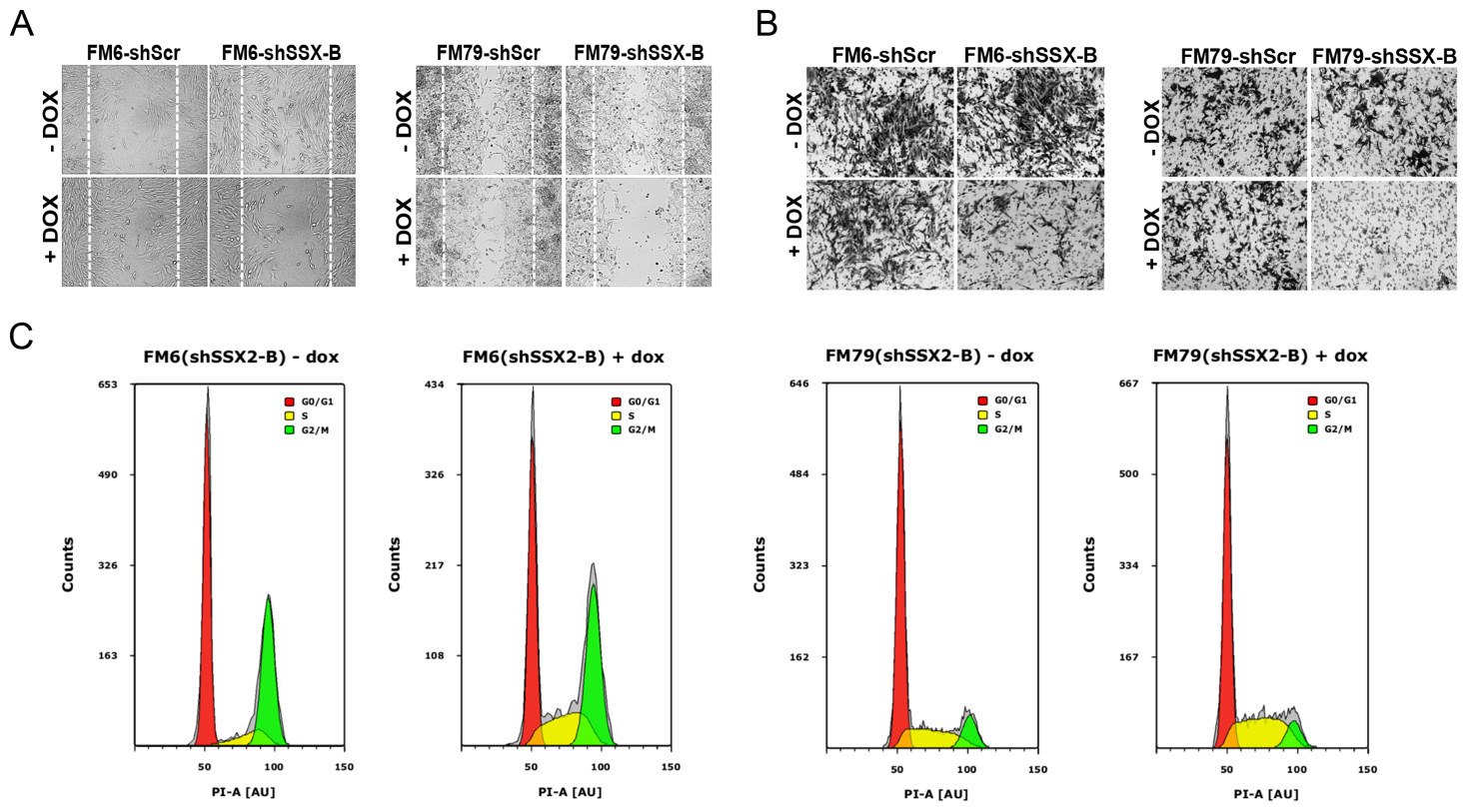


**Figure S5. SSX knockdown reduces expression of genes supporting migration and invasion.** Functional enrichment analysis using HOMER revealed that genes involved in several metastases-associated biological processes such as focal adhesion, integrin-mediated cell adhesion and ECM-receptor interaction was repressed in the FM6, but not FM79, cell line, in response to SSX silencing. Error bars represent SD (*n* = 3). (****) *P* < 0.0001; (***) *P* < 0.001; (**) *P* < 0.01; (*) *P* < 0.05; (NS) not significant (*P* > 0.05).


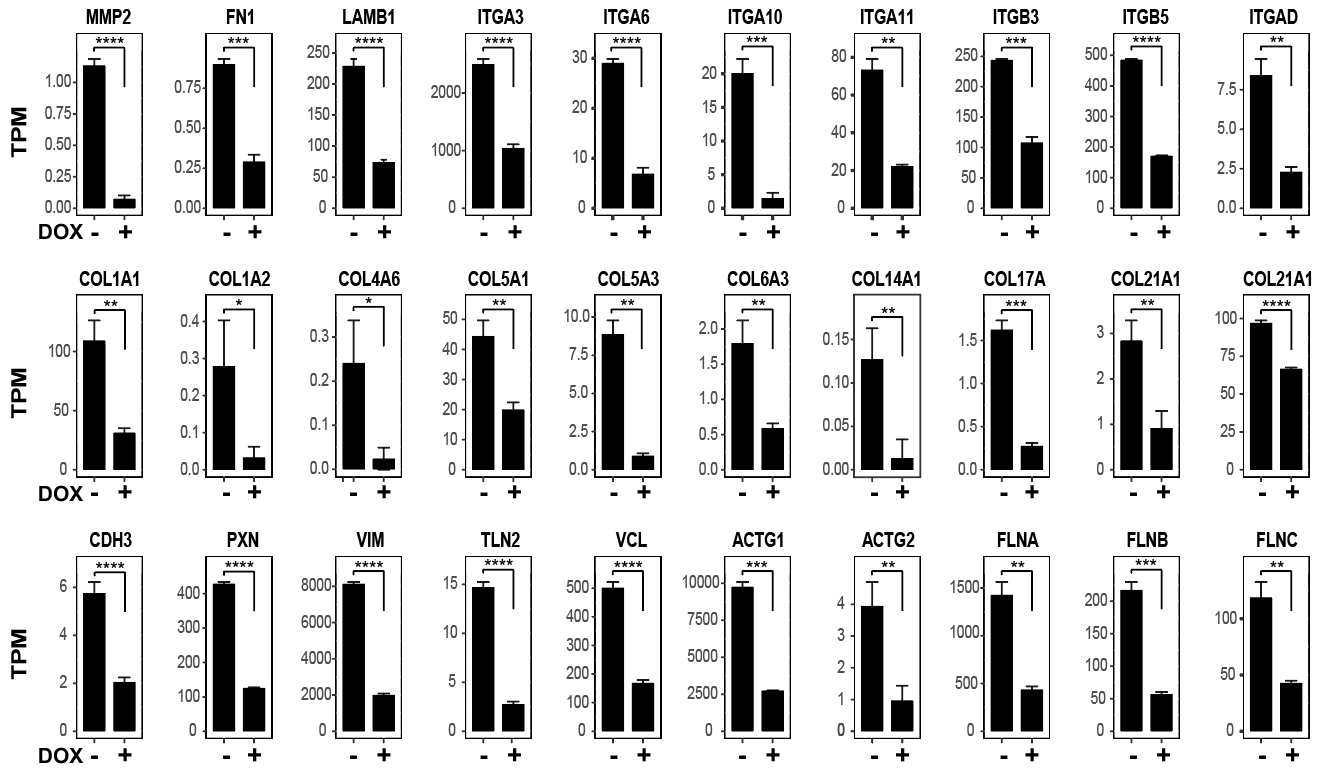

Supplement: Supplementary file 1 [file DataSheet_1.docx]
